# Hepatitis B Stigma and Knowledge among Vietnamese in Ho Chi Minh City and Chicago

**DOI:** 10.1155/2016/1910292

**Published:** 2016-12-22

**Authors:** Lan Dam, Anita Cheng, Phuong Tran, Shirley S. Wong, Ronald Hershow, Sheldon Cotler, Scott J. Cotler

**Affiliations:** ^1^University of Illinois at Chicago, Chicago, IL, USA; ^2^Loyola University Medical Center, Maywood, IL, USA; ^3^RUSH University Medical Center, Chicago, IL, USA; ^4^DePaul University, Chicago, IL, USA

## Abstract

Stigma regarding viral hepatitis and liver disease has psychological and social consequences including causing negative self-image, disrupting relationships, and providing a barrier to prevention, testing, and treatment. The aim of this study was to characterize and compare HBV knowledge and stigma in Vietnamese in Ho Chi Minh City and Chicago and to begin to evaluate the cultural context of HBV stigma.* Methods*. A written survey including knowledge questions and a validated HBV stigma questionnaire was distributed to Vietnamese in Ho Chi Minh City and Chicago. 842 surveys from Ho Chi Minh City and 170 from Chicago were analyzed.* Results*. Vietnamese living in Chicago had better understanding of HBV transmission and that HBV can cause chronic infection and liver cancer. Vietnamese in Chicago had higher stigma scores on a broad range of items including guilt and shame about HBV and were more likely to feel that persons with HBV can bring harm to others and should be isolated.* Conclusions*. Vietnamese in Ho Chi Minh City and Chicago have knowledge deficits about HBV, particularly regarding modes of transmission. Persons in Ho Chi Minh City expressed lower levels of HBV stigma than Vietnamese living in Chicago, likely reflecting changing cultural attitudes in Vietnam. Culturally appropriate educational initiatives are needed to address the problem of HBV stigma.

## 1. Introduction

Hepatitis B virus (HBV) infection is endemic in Asia and is common in Asian immigrants to the United States. In Vietnam, the HBsAg seroprevalence is in the range of 11.4% to 15.3% [1, 2] and the prevalence is 8.8% to 13.8% in Vietnamese Americans in California [3, 4]. Hepatitis B is a leading cause of chronic liver disease, cirrhosis, and hepatocellular carcinoma (HCC) in Asia and HCC is the most common cause of cancer death in Vietnam and the second most frequent cause of cancer mortality in China [5]. While the physical health implications of HBV have been studied extensively, less attention has been given to HBV-related stigma, which can affect social interactions, psychological well-being, and the willingness to seek medical care.

Health-related stigma can be conceptualized as an adverse social judgment resulting in exclusion, rejection, blame, or devaluation. Stigma can damage self-esteem, have an impact on social and economic status, lead to discrimination, and adversely affect family members [6, 7]. Persons who perceive stigma anticipate adverse outcomes such as disrupted relationships and social ostracism [8]. Affected individuals internalize social prejudices, which may result in negative self-image and concern that they might lose the respect of their family or community [8]. Illnesses carrying stigma have the potential to disrupt the harmony of family relationships, which are a central focus in Asian societies [9]. The collectivist nature of Asian cultures and the critical role of personal relationships can result in susceptibility to the negative influences of stigma among persons of Asian background.

A small number of studies reported to date on HBV stigma provide evidence that it has important behavioral and psychological consequences. As an example, a survey of Chinese Canadians found persons with higher levels of stigma were less likely to be have been screened for HBV [10]. A publication from Malaysia evaluated the concerns of persons with HBV among a study population that was comprised largely of Chinese immigrants. More than half of participants reported that they were worried about spreading the infection to family and friends, and over a quarter were unable to enjoy daily activities since their diagnosis [11]. In a previous investigation, we developed and validated an HBV stigma instrument and piloted it in Chinese and English [12]. A substantial proportion of immigrants in an urban Chinatown neighborhood reported stigma related to HBV, and there was particular concern that persons with HBV pose an infectious risk. Similar to other studies [9, 13], the respondents showed knowledge deficits regarding HBV, most notably reflecting a lack of understanding of how the infection is transmitted.

It is not known whether the observations regarding HBV stigma in Chinese immigrants are relevant to other Asian groups or if HBV stigma differs between immigrants to the United States and persons residing in Asia. The aim of this study was to assess knowledge and attitudes about HBV in Ho Chi Minh City and to compare data from Vietnam to the views and understanding of HBV among Vietnamese immigrants living in Chicago.

## 2. Materials and Methods

The study was approved by the Institutional Review Board at the University of Illinois at Chicago and the Human Subjects Committees at the University of Medicine and Pharmacy in Ho Chi Minh City, Vietnam. The previously validated HBV stigma questionnaire [12] was translated from English to Vietnamese along with demographic and HBV knowledge items by a professional translator and then back translated into English by a Vietnamese speaking healthcare professional to ensure accuracy. Minor modifications were made to the survey to make it appropriate for Vietnamese participants.

### 2.1. US Study Site

A bilingual investigator distributed questionnaires to a convenience sample of Vietnamese Americans who presented for a routine office visit to a Vietnamese speaking primary care physician (P. T.) in Chicago, IL. The bilingual investigator was available to answer participant's questions about the survey. Three hundred persons were approached to fill out the survey between October 2011 and March 2012. Two hundred twenty-six persons agreed to take the survey (75%) and 56 participants were excluded because they failed to complete more than 10 items, leaving data from 170 surveys for analysis.

### 2.2. Vietnamese Study Sites

Participants were recruited at two hospitals affiliated with the University of Medicine and Pharmacy in Ho Chi Minh City. The participants consisted of a convenience sample of patients and family members of patients seeking care at The People's Hospital of Gia Dinh, located in Binh Thanh district, and the Children's Hospital 2, located in District 1 of Ho Chi Minh City. The same investigator who administered the survey in Chicago approached participants at large queuing areas in the hospitals, including but not limited to patients waiting for clinic appointments, laboratory testing, payment, and other hospital facilities. Patients who were identified as minors or expectant mothers were not included. Participants were provided a room to complete the survey in private. One thousand two hundred surveys were distributed from January 2013 to October 2013. Nine hundred twenty-eight surveys were filled out (77%) including 722 from Children's Hospital 2 and 206 from The People's Hospital of Gia Dinh. Eighty-six participants were excluded because they failed to complete more than 10 items, leaving 842 surveys for analysis.

### 2.3. Statistical Analysis

Descriptive statistics were used to check for erroneous entries. A weighted total HBV stigma score was calculated by numbering responses for each stigma item 1–4 (low to high stigma) and summing the result for each participant such that higher values indicated higher levels of stigma. For analysis pertaining to individual stigma items, responses were dichotomized as stigma (yes/no). An HBV knowledge score was calculated as the sum of the number of correct responses to the 12 HBV knowledge questions.

Associations were evaluated between demographic variables and knowledge and stigma scores. Demographic data and knowledge and stigma scores were compared between Vietnamese in Ho Chi Minh City and Chicago. Normally distributed continuous variables were analyzed by Student's* t*-test or ANOVA. Chi-square testing was used to compare categorical data. The relationship between continuous variables was assessed by Pearson's correlation coefficients. All statistical analyses were performed using SPSS, version 19.0 (Chicago, IL, USA).

## 3. Results

### 3.1. Demographics

The study population in Ho Chi Minh City consisted of 842 persons with a mean age of 36 ± 11 years (Table 1), 60% of whom were women. A majority of participants were married (86%) and 76% were employed. Fifty-seven percent of participants had at least a high school education and, among those, 42% had university or postgraduate education. A majority of participants resided in southern Vietnam. While only 44% of the population reported having been tested for HBV, 65% denied being an HBV carrier, and 4% identified as carriers. Among those who reported that they had not been tested for HBV, only 72% stated that they would be willing to do so. Fourteen percent of the population had family members who were HBV carriers. Only 33% percent of participants recalled being vaccinated for HBV.

The study population in Chicago consisted of 170 participants with a mean age of 47 ± 16 years, and 54% were women (Table 1). Seventy-seven percent were married and 67% were employed. A majority of the population had a high school education (86%), and, among those, 42% had university or postgraduate education. Eighty-seven percent of participants had health insurance. While 44% of the population reported that they had been tested for HBV, 57% denied being an HBV carrier and 9% reported that they were carriers. Among those who have not been tested, only 55% stated that they would be willing to be tested for HBV. Fourteen percent had a family member who was an HBV carrier. Forty-eight percent of the population reported that they had been vaccinated against HBV.

Vietnamese living in Chicago were older (*p* < 0.001) and less likely to be married (*p* = 0.004) than participants in Ho Chi Minh City (Table 1). A higher proportion of participants in Chicago had at least a high school education (*p* < 0.001). Vietnamese living in Chicago reported a higher rate of HBV vaccination (*p* < 0.001) and a larger proportion identified as HBV carriers (*p* = 0.005). There were no gender differences between the two groups and they reported similar rates of previous HBV testing, willingness to be tested, and prevalence of carriers in the family.

#### 3.1.1. HBV Knowledge

As shown in Table 2, 90% or more of participants in Ho Chi Minh City were correct in understanding that HBV is preventable by vaccination, that HBV can cause advanced liver disease (cirrhosis) and liver cancer, and that the infection can be diagnosed by a blood test. In contrast, substantial knowledge deficits were present regarding transmission and chronicity of the infection. Fifty-five percent of participants did not understand that HBV can be spread by sexual intercourse, 55% had the mistaken impression that HBV can be spread by sharing eating utensils, and 31% thought that the disease could be contracted by eating shellfish. Moreover, 48% did not know that HBV can cause a lifelong infection.

Among participants in Ho Chi Minh City, HBV total knowledge scores were associated with demographic factors. Persons who had at least a high school education had higher total knowledge scores than those who did not complete high school (*p* = 0.001). Participants who reported that they had been previously tested for HBV (*p* < 0.001), those who were willing to be tested (*p* = 0.002), participants who identified as an HBV carrier (*p* < 0.001), and persons with an HBV carrier in the family (*p* < 0.001) had higher total knowledge scores than those who did not. There was no association between total HBV knowledge score and age, sex, marital status, or employment status.

More than ninety percent of Vietnamese participants living in Chicago understood that HBV is preventable by vaccination, is only identifiable by a blood test, and can be treated with prescription medications (Table 2). Eighty-five percent understood that HBV can cause liver cancer and 72% were aware that it can cause a lifelong infection. Similar to the participants in Ho Chi Minh City, those in Chicago showed knowledge deficits with regard to means of HBV transmission. Thirty-one percent did not know that HBV could be spread by sexual intercourse, 32% thought transmission can occur through eating utensils, and 46% felt it could be contracted by eating shellfish.

Mean total knowledge scores were similar between Vietnamese living in Chicago (9.1 ± 2.0) and persons in Ho Chi Minh City (9.0 ± 1.7) (*p* = 0.892). The Chicago group showed higher levels of knowledge regarding some aspects of HBV transmission (Table 2). Compared to participants from Ho Chi Minh City, those living in Chicago had higher rates of understanding that HBV is spread by sexual intercourse (*p* < 0.001) and that HBV is not spread by sharing eating utensils (*p* = 0.001). The Vietnamese in Chicago more frequently recognized that HBV can cause lifelong infection (*p* < 0.001). In contrast, participants in Ho Chi Minh City had a better understanding that HBV is not contracted by eating shellfish (*p* < 0.001) and that it can cause liver cancer (*p* = 0.042). The two groups showed similar levels of knowledge on all other items.

#### 3.1.2. HBV Stigma

Among respondents in Ho Chi Minh City, 61% felt that persons with HBV put others at risk and 43% indicated that they should avoid close contact such as hugging or kissing (Table 3). Fifty-three percent felt that persons with HBV feel that they bring trouble to their family. In contrast, 87% responded that persons with HBV should not be isolated and only 28% felt that they were undesirable as a spouse. There was a limited association between HBV and shame with only 17% of participants in Ho Chi Minh City surmising that those infected with HBV feel ashamed and 22% indicated that HBV might be viewed as a shameful sexually transmitted disease. Only about 20% felt that persons with HBV might be discriminated against in school or work in Vietnam and only 8% believed that HBV carriers might be denied healthcare in Vietnam.

Cumulative stigma scores were associated with demographic factors and total knowledge scores in the Ho Chi Minh City cohort. There was a correlation between older age and higher stigma scores (*r* = 0.224, *p* < 0.001). Persons who were married had higher cumulative stigma scores (*p* < 0.001) and were also older (*p* < 0.001) than those who were single. Respondents with less than a high school education reported more HBV stigma than those who had more education (*p* < 0.001). Persons who reported that they had not been tested or did not know if they had been tested for HBV had a higher cumulative stigma score than those who reported that they had been tested (*p* = 0.003). Similarly, respondents who were unwilling to be tested had higher stigma scores (*p* = 0.009). Individuals who reported that they were not carriers or did not know their carrier status had higher levels of stigma (*p* = 0.011), as did those who did not have a carrier in the family (*p* = 0.025). There was a marginal positive correlation between total knowledge score and cumulative stigma score (*r* = 0.092, *p* = 0.007). There was no association between cumulative stigma score and sex, employment status, or insurance status.

Among the Vietnamese living in Chicago, 52% thought that persons with HBV feel that they bring trouble to their family, 49% indicated that they can bring harm to others, and 44% thought that they should avoid close contact such as hugging or kissing (Table 3). In contrast, 63% responded that persons with HBV should not be isolated and only 25% felt that they were undesirable as a spouse. Twenty-nine percent of Vietnamese participants in Chicago felt that persons infected with HBV feel ashamed and 30% indicated that HBV might be viewed as a shameful sexually transmitted disease. Twenty-nine percent felt that persons with HBV might be discriminated against in school or work in Vietnam and 21% believed that HBV carriers might be denied healthcare in Vietnam.

Vietnamese living in Chicago who were tested for HBV had lower cumulative stigma scores than those who reported that they were not tested (*p* = 0.041). Unlike in the Ho Chi Minh City group, there were no other associations between demographic variables and cumulative stigma score or total knowledge score in the Chicago cohort.

Vietnamese living in Chicago had higher total stigma scores than the Ho Chi Minh City group (*p* < 0.001), reflecting their feelings about a broad range of items (Table 3). Vietnamese in Chicago more frequently responded that persons with HBV feel ashamed about having the disease (*p* < 0.001), feel guilty about having HBV (*p* < 0.001), and think that HBV is a shameful sexually transmitted disease (*p* = 0.011). Compared to the group from Ho Chi Minh City, the Chicago participants were more likely to feel that persons with HBV can bring harm to others (*p* = 0.005), should be isolated (*p* = 0.001), and cannot be trusted as a friend (*p* = 0.001). The Vietnamese in Chicago were more likely to think that HBV-infected persons living in Vietnam would experience discrimination at school (*p* = 0.004) and work (*p* = 0.023) and might be denied healthcare (*p* < 0.0001) compared to participants in Ho Chi Minh City. In contrast, the Vietnamese in living in Chicago were less likely to feel that persons with HBV put others at risk for HBV than participants in Ho Chi Minh City (*p* < 0.001).

## 4. Discussion

The current study provides new and important information about HBV knowledge and stigma. Vietnamese in Ho Chi Minh City and Chicago showed substantial knowledge deficits regarding how HBV is transmitted that can put uninfected persons at risk and contribute to stigma. Most strikingly, more than half of respondents from Ho Chi Minh City were not aware that HBV can be spread by sexual intercourse and a majority thought that HBV could be spread by sharing eating utensils. There were notable similarities and differences in HBV stigma between participants in Ho Chi Minh City and in Chicago. A similar proportion of participants in both groups expressed concern that persons with HBV bring trouble to their family and that they should avoid close contact with others. Vietnamese in Chicago more frequently attributed feelings of shame and guilt to those with HBV, while participants in Ho Chi Minh City more often expressed that infected individuals put others at risk for HBV. Conflicting attitudes about HBV were identified that reflect the complexity of HBV stigma. Many participants in both groups felt that persons with HBV should avoid close contact with others. In contrast, a majority of respondents from Ho Chi Minh City and Chicago felt that people with HBV should not be isolated and a majority disagreed that having HBV makes someone undesirable as a spouse. The apparent contradictory views on HBV stigma might reflect differing perspectives when persons with HBV are viewed as individuals versus an impersonal group with an infectious disease that poses a threat of transmission.

Persons in Vietnam and Vietnamese immigrants to the US are exposed to new ideas and belief systems that challenge traditional values. Even as societal changes are occurring, traditional values have an impact on how people perceive illness and how they respond to those who are affected. Illness, including HBV, is traditionally viewed in a different cultural context in Asian cultures, where infectious diseases may be considered the responsibility of the affected person [14]. Family and personal relationships are revered and shame and guilt associated with illness may be shared by family members [15].

Comparing HBV stigma between Vietnamese in Ho Chi Minh City and Chicago provides insight into the impact of changing cultural influences on views on HBV. The Vietnamese in Ho Chi Minh City expressed more progressive perspectives about HBV than those residing in Chicago. They reported lower HBV stigma scores regarding bringing harm to others, guilt, and shame than their counterparts in the US. Furthermore, Vietnamese living in Chicago felt that people in Vietnam with HBV were more likely to face discrimination at work and school than did participants living in Vietnam. Although Vietnamese in Chicago were living in a Western society and as a group had received more education, their higher levels of stigma on a range of issues might be related in part to their older age and the likelihood that they left Vietnam rooted in traditional values and perceptions. A recent study performed in Vietnam found that HIV infected injection drug users experienced support and concern from their families, even as they faced stigma and discrimination in their communities [16]. The findings regarding HIV suggest that Vietnamese living in Chicago also might express higher levels of HBV stigma than persons in Ho Chi Minh City due to a decrease in extended family connections and social support in the US.

Both Vietnamese groups expressed stigma with regard to HBV bringing trouble to the family. In Asian cultures, illness may be viewed as a shared experience and having an infection can bring shame to the patient as well as to the family [14]. Upsetting the balance and harmony in the family is viewed as a shameful act. Shame and guilt play an important cultural role and can limit information seeking about infectious diseases such as HBV, providing a potential barrier to assimilating knowledge about the disease. Stigma leads to avoidance behaviors including resistance to seeking information about health, a reluctance to disclose personal information to social networks, and caution about seeking treatment and adhering to treatment protocols [14, 15]. Negative feelings about oneself and associated stigma are associated with poorer health outcomes [17]. Asian fatalism might provide another barrier to seeking medical care including vaccination, testing and treatment [15], since life may be believed to be unpredictable and that people have limited control over their own destiny. In the current study, self-reported testing and vaccination rates were low among both Vietnamese groups. Education and resources to perform testing and vaccination could reduce HBV stigma by empowering individuals to address uncertainty about HBV in their own lives and to mitigate risk through prevention or through encouraging treatment when indicated. Educating physicians also will be important as previous studies have shown low levels of knowledge about risk factors and screening for HBV among residents in training and practicing physicians [18, 19].

Misinformation, which was particularly evident with regard to how HBV is transmitted, can contribute to stigma and isolation. As an example, a common misconception was that HBV can be spread by sharing eating utensils, which could interfere with the custom of sharing food and is at odds with traditional social practices and the collectivistic nature of Asian society. Data from the current study indicate that educational efforts are needed to improve HBV knowledge, particularly with regard to transmission. However, general education about HBV is unlikely to be sufficient to change attitudes about the disease. In Vietnam, higher total knowledge scores were weakly associated with higher levels of stigma, indicating that there might be a fine line between alarming people regarding the risks of the disease and empowering them with knowledge. Educational efforts should be developed in the context of cultural views including the potential impact of disclosing an illness on family and social networks and educational initiatives could be informed by data regarding HBV stigma.

Our previous study assessed HBV knowledge and stigma in Chinese immigrants to the US [12]. The pattern of knowledge deficits observed among Vietnamese in the current study regarding HBV transmission, including misconceptions about spread through eating utensil, eating shellfish, and underestimating the role of sexual intercourse, was similar to that previously observed in Chinese Americans. Stigma also was prominent among Chinese Americans, with over 60% of respondents believing that HBV put others at risk and 36% feeling that HBV brought trouble to the family [12]. These findings suggest commonalities among Asian groups with regard to HBV stigma.

Weaknesses of this study include the use of convenience samples of persons presenting for healthcare in Ho Chi Minh City and Chicago. The source of the participants and the relatively small number of respondents in the Chicago sample raise the possibility that the data are not completely representative of the views of Vietnamese regarding hepatitis B. Strengths of the study include the use of a validated stigma survey that was translated into Vietnamese with minor modifications for cultural relevance. The same bilingual investigator administered all surveys in Ho Chi Minh City and Chicago and provided consistent instructions and answers to participant's questions about the survey when needed in both locations. The sample size in Ho Chi Minh City was large and the study was the first to compare attitudes about HBV among persons living in Asia and Asian immigrants to the US.

## 5. Conclusions

**Figure 1:**
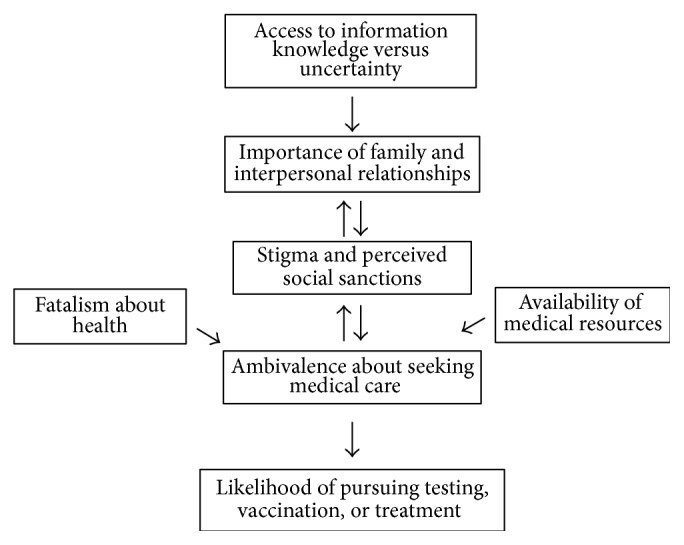
Multiple factors that have an impact on how HBV infection is perceived, which can affect psychological well-being and social interactions and serve as a barrier to seeking testing, vaccination, and treatment.

The current study begins to identify some of the complex factors that have an impact on how persons in Vietnam and Vietnamese Americans view illnesses such as HBV, which can have an impact on psychological well-being and social interactions. Stigma can lead to negative stereotypes and labels, producing expectations of social sanctions. Uncertainty and stigma can have a deleterious effect on family relationships and social networks. These cultural factors have the potential to interfere with efforts to educate and treat people with HBV. Misinformation, uncertainty, and stigma create barriers to testing, vaccination, and treatment, while a knowledge deficit about the risk of sexual transmission can contribute to spread of the disease. Stigma and associated cultural values can contribute to avoidance of health-related issues and reduce the probability of seeking care. Fatalism about health and availability of medical resources can further contribute to ambivalence about seeking medical care (Figure 1). Understanding cultural values, providing education at the community level to address specific knowledge gaps such as modes of HBV transmission and the protective effect of vaccination, and humanizing persons with HBV provide starting points to address issues of HBV stigma. Physicians will need to play a leading role in educating their patients and affecting change in attitudes about HBV.

## Figures and Tables

**Table 1:** Demographic data.

	Vietnamese in Chicago (*n* = 170)		Vietnamese in Ho Chi Minh City (*n* = 842)		*p* value
	Number of valid responses		Number of valid responses		
Age (mean ± SD years)	170	47 ± 16	842	36 ± 11	<0.001
Sex (% male/female)	169	46%/54%	842	40%/60%	0.191
Marital status (% married/single)	170	77%/23%	842	86%/14%	0.004
Education (%<high school/≥high school)	169	14%/86%	842	43%/57%	<0.001
Recalls testing for HBV (%)	165	44%	842	44%	0.966
Self-identified as HBV carrier (%)	166	9%	840	4%	0.005
HBV carrier in the family (%)	169	14%	840	14%	0.926
Vaccinated for HBV (%)	166	48%	842	33%	<0.001

**Table 2:** Hepatitis B knowledge. Participants were asked to indicate whether the following statements about hepatitis B are true or false.

Statement about hepatitis B	Vietnamese in Chicago *n* (%) correct	Vietnamese in Ho Chi Minh City *n* (%) correct	*p* value
Is preventable by vaccination	161/170 (95%)	811/842 (96%)	0.325
Can be treated with prescription medications	155/170 (91%)	753/842 (89%)	0.494
Can be spread by sexual intercourse	117/169 (69%)	383/842 (45%)	<0.001
Can be spread by blood	139/170 (82%)	639/842 (76%)	0.098
Can be spread during childbirth	122/170 (72%)	656/842 (78%)	0.083
Can be spread by eating raw shellfish	92/170 (54%)	578/841 (69%)	<0.001
Can be spread by sharing eating utensils	116/170 (68%)	379/842 (45%)	<0.001
Can cause lifelong infection	122/170 (72%)	441/842 (52%)	<0.001
Can cause advanced liver disease (cirrhosis)	150/170 (88%)	778/842 (92%)	0.073
Can cause liver cancer	145/170 (85%)	762/842 (91%)	0.042
Can be spread by someone who looks healthy	137/170 (81%)	632/842 (75%)	0.124
Carriers can only be identified by a blood test	159/170 (94%)	805/842 (96%)	0.245

**Table 3:** Responses to stigma items. Respondents were asked to provide their perceptions about people who are hepatitis B carriers.

	Vietnamese in Chicago					Vietnamese in Ho Chi Minh City					
	Number of valid responses	Strongly agree	Agree	Disagree	Strongly disagree	Number of valid responses	Strongly agree	Agree	Disagree	Strongly disagree	*p*value^*∗*^
(i) Feel ashamed about having HBV	169	11%	18%	60%	11%	842	1%	16%	78%	5%	<0.001
(ii) Feel that they bring trouble to their family	170	14%	38%	42%	6%	842	1%	52%	45%	2%	0.839
(iii) Feel guilty about having HBV	170	9%	20%	61%	9%	842	1%	16%	78%	5%	<0.001
(iv) Put others at risk for HBV	170	13%	24%	54%	9%	842	2%	59%	37%	2%	<0.001
(v) Should avoid close contact with others such as kissing or hugging	170	16%	28%	46%	10%	842	2%	41%	54%	3%	0.897
(vi) Should not be isolated	170	22%	41%	30%	7%	842	8%	79%	11%	2%	<0.001
(vii) May be viewed by others as having a shameful sexually transmitted disease	168	8%	22%	61%	9%	842	1%	21%	73%	5%	0.011
(viii) Can be trusted not to bring harm to others	170	8%	43%	39%	10%	842	3%	60%	35%	2%	0.005
(ix) Can be trusted as friends	170	14%	58%	24%	4%	842	4%	83%	12%	1%	<0.001
(x) Are viewed as undesirable as a husband or wife	170	8%	17%	64%	11%	842	1%	27%	69%	3%	0.486
(xi) Might be discriminated against at school in the Vietnam	170	7%	22%	60%	11%	842	1%	19%	75%	5%	0.004
(xii) Might be discriminated against at work in the Vietnam	170	8%	21%	60%	11%	842	1%	20%	74%	5%	0.023
(xiii) Might be denied healthcare in Vietnam	169	6%	15%	66%	13%	842	1%	7%	82%	10%	<0.001

^*∗*^For analysis pertaining to individual stigma items, responses were dichotomized as stigma (yes/no).
